# CDK4/6 inhibitors, PI3K/mTOR inhibitors, and HDAC inhibitors as second-line treatments for hormone receptor-positive, HER2-negative advanced breast cancer: a network meta-analysis

**DOI:** 10.1186/s12885-023-11290-7

**Published:** 2023-08-29

**Authors:** Danyang Ji, Yang Luo, Jiayu Wang, Shanshan Chen, Bo Lan, Fei Ma, Binghe Xu, Ying Fan

**Affiliations:** Department of Medical Oncology, National Clinical Research Center for Cancer/Cancer Hospital, National Cancer Center, Chinese Academy of Medical Sciences & Peking Union Medical College, Beijing, 100021 China

**Keywords:** CDK4/6 inhibitors, Hormone receptor-positive, Metastatic breast cancer, Network meta-analysis

## Abstract

**Background:**

This study sought to compare the benefits and safety of agents including Cyclin-dependent kinase 4/6 (CDK4/6) inhibitors, phosphoinositide 3-kinase (PI3K)/mammalian target of rapamycin (mTOR) inhibitors, and histone deacetylase (HDAC) inhibitors as second-line treatments for these patients by conducting a comprehensive systematic review and network meta-analysis.

**Methods:**

The Medline, Embase and Cochrane Library databases were searched for randomized trials comparing CDK4/6 inhibitors, PI3K/mTOR inhibitors, or HDAC inhibitors vs. placebo with the addition of exemestane or fulvestrant as second-line treatments in patients with HR + advanced breast cancer up to December 16, 2021. Outcomes of interest were progression-free survival (PFS), overall response rate (ORR), overall survival (OS), clinical benefit rate (CBR), and grade 3–4 adverse drug events (ADEs). The present study was conducted according to the Cochrane Collaboration and PRISMA statements. The overall effect was pooled using the random effects model.

**Results:**

Seventeen studies with a total of 9,100 participants were included in the current study. Compared with placebo plus fulvestrant, PFS was significantly improved by CDK4/6 inhibitor plus fulvestrant, mTOR inhibitor plus fulvestrant, mTOR inhibitor plus exemestane, and PI3K inhibitor plus fulvestrant, but not HDAC inhibitor plus exemestane. While mTOR inhibitor plus exemestane was the best regimen (SUCRA value 89.5%), the mTOR inhibitor plus exemestane regimen induced more severe adverse events (SAEs) than the HDAC inhibitor plus exemestane regimen [OR, 95% CI: 2.40 (1.40–4.10)].

**Conclusion:**

mTOR inhibitor and CDK4/6 inhibitor-based regimens demonstrated superior clinical efficacy and comparable safety profiles as second-line treatment in patients with HR-positive, HER2-negative advanced breast cancer.

**Supplementary Information:**

The online version contains supplementary material available at 10.1186/s12885-023-11290-7.

## Introduction

In 2020, an estimated 2.3 million cases of breast cancer were newly diagnosed worldwide, accounting for approximately 25% of female malignant tumors and overtaking lung cancer as the most common malignancy [1]. Among these breast cancer cases, hormone receptor (HR)-positive, human epidermal growth factor receptor 2 (HER2)-negative tumors are the predominant subtype, affecting more than 75% of all cases [2]. In recent years, the accumulation of clinical evidence has changed the treatment mode of HR-positive, HER2-negative advanced breast cancer from endocrine therapy to endocrine therapy combined with targeted therapies as the first-line treatment [3]. However, endocrine monotherapy is still considered the standard and used for the first-line treatment of HR-positive, HER2-negative advanced breast cancer in many cases [4] because of various factors, such as treatment concepts, the accessibility of pharmaceuticals, and economic conditions. However, nearly all patients acquire resistance to therapy [5], and the need for second-line therapies that can potentially improve the prognosis of patients with the onset of endocrine resistance is urgent.

The cyclin D–cyclin-dependent kinase (CDK) 4/6–retinoblastoma pathway is frequently dysregulated in HR-positive, HER2-negative breast cancer [6] and is implicated in resistance to endocrine monotherapy [7]. Several phase 3 trials have demonstrated the utility of combining a CDK4/6 inhibitor, including ribociclib, palbociclib, abemaciclib and dalpiciclib, with endocrine therapy as a second-line treatment for HR-positive, HER2-negative advanced breast cancer [8–13]. In addition, aberrant signaling through the phosphatidylinositol 3-kinase (PI3K)–mammalian target of rapamycin (mTOR) signaling pathway also plays a critical role in endocrine resistance [14], which can be attenuated by PI3K/mTOR inhibitors, such as alpelisib and everolimus. Epigenetic modification alters gene expression and contributes to endocrine therapy resistance, which may be reversed by epigenetic modifiers, such as histone deacetylase (HDAC) inhibitors [15–17]. In recent decades, both PI3K/mTOR inhibitors and HDAC inhibitors (chidamide and entinostat) have shown clinical benefits as second-line treatments in clinical trials at the onset of endocrine resistance [18–20].

However, these agents have not been directly compared, which makes it difficult to provide information for the selection of treatment regimens in clinical practice. Thus, the present study sought to clarify this issue by conducting a comprehensive systematic review and network meta-analysis to compare the benefits of CDK4/6 inhibitors, PI3K/mTOR inhibitors, and HDAC inhibitors as second-line treatments in patients with HR-positive, HER2-negative advanced breast cancer based on clinical outcomes.

## Methods

### Data sources

An electronic search of the PubMed (http://www.ncbi.nlm.nih.gov/PubMed/), EMBASE (http://store.elsevier.com/embase) and Cochrane Library CENTRAL (https://www.cochranelibrary.com/) databases was performed for studies published up to December 16, 2021. Search terms were defined considering participants (“advanced” AND “hormone receptor-positive” AND “breast cancer”) and interventions (“hormone therapy” OR “CDK4/6” OR “CDK4”, “CDK6” OR “PI3K” OR “mTOR” OR “HDAC”) to guarantee the high sensitivity of the electronic search and were further restricted to clinical trials and studies in humans. We also hand searched the bibliographies of recently published meta-analyses of related reagents [21–23]. The full texts of relevant citations from all identified results were inspected and analyzed. From the main search results, relevant references to the inputted key words were also searched and reviewed accordingly. The present study was conducted according to the Cochrane Collaboration and Preferred Reporting Items for Systematic Review and Meta-Analyses (PRISMA) statement.

### Inclusion and exclusion criteria

Relevant studies were included based on prospectively established inclusion criteria as follows: the studies were in patients with HR-positive, HER2-negative advanced breast cancer; the drugs were employed as second-line treatment; the studies compared CDK4/6 inhibitors, PI3K/mTOR inhibitors, or HDAC inhibitors vs. placebo and the addition of exemestane or fulvestrant; the studies were randomized, controlled trials; and the primary publication was in English. For PI3K inhibitors, only studies in patients with PIK3CA-mutated cancer were included.

The exclusion criteria were as follows: the study was an animal experiment, research progress, drug mechanism discussion, medication guidance, scheme interpretation, or systematic evaluation; the literature did not completely report the necessary research methods and results; and the study language was not English. For duplicated reported trials, only the longest report and follow-up data were included. Two independent reviewers read the literature and selected the studies included for the analysis.

### Data extraction and quality assessment

A standard data collection form was designed to arrange the extracted data of interest. Information was extracted by two independent reviewers, including study name, published years and journals, follow-up periods, number and age of participants, type and dosage of medication, control agent, and clinical outcomes (PFS, ORR, CBR and safety parameters). The methodological quality of each study was assessed separately. The quality of the data included in the present study was assessed using the Cochrane Collaboration’s tool.

### Statistical analysis

Categorical outcomes were reported as numbers, and the odds ratio (RR) and corresponding 95% confidence intervals (CIs) for the outcomes were calculated for each trial. Heterogeneity for the treatment effects among each selected study was tested with *χ2* tests, and the extent of the heterogeneity between studies was assessed with *I*^*2*^. A network meta-analysis with both fixed and random effect models was performed by using Stata with the mvmeta package to assess the treatment effects for the clinical outcomes and the interstudy variances. The network plots were drawn using the network package based on the Stata software. We ranked the treatment regimens according to the surface under the cumulative ranking curve (SUCRA). The SUCRA was expressed as a percentage, where 100% indicated that a treatment was ensured to be the best, and 0% indicated that a treatment was ensured to be the worst. A higher SUCRA percentage indicated that a treatment had a higher rank among the network treatment regimens [24]. Statistical tests with P < 0.05 were considered significant.

## Results

### Characteristics of the included studies

**Fig. 1:**
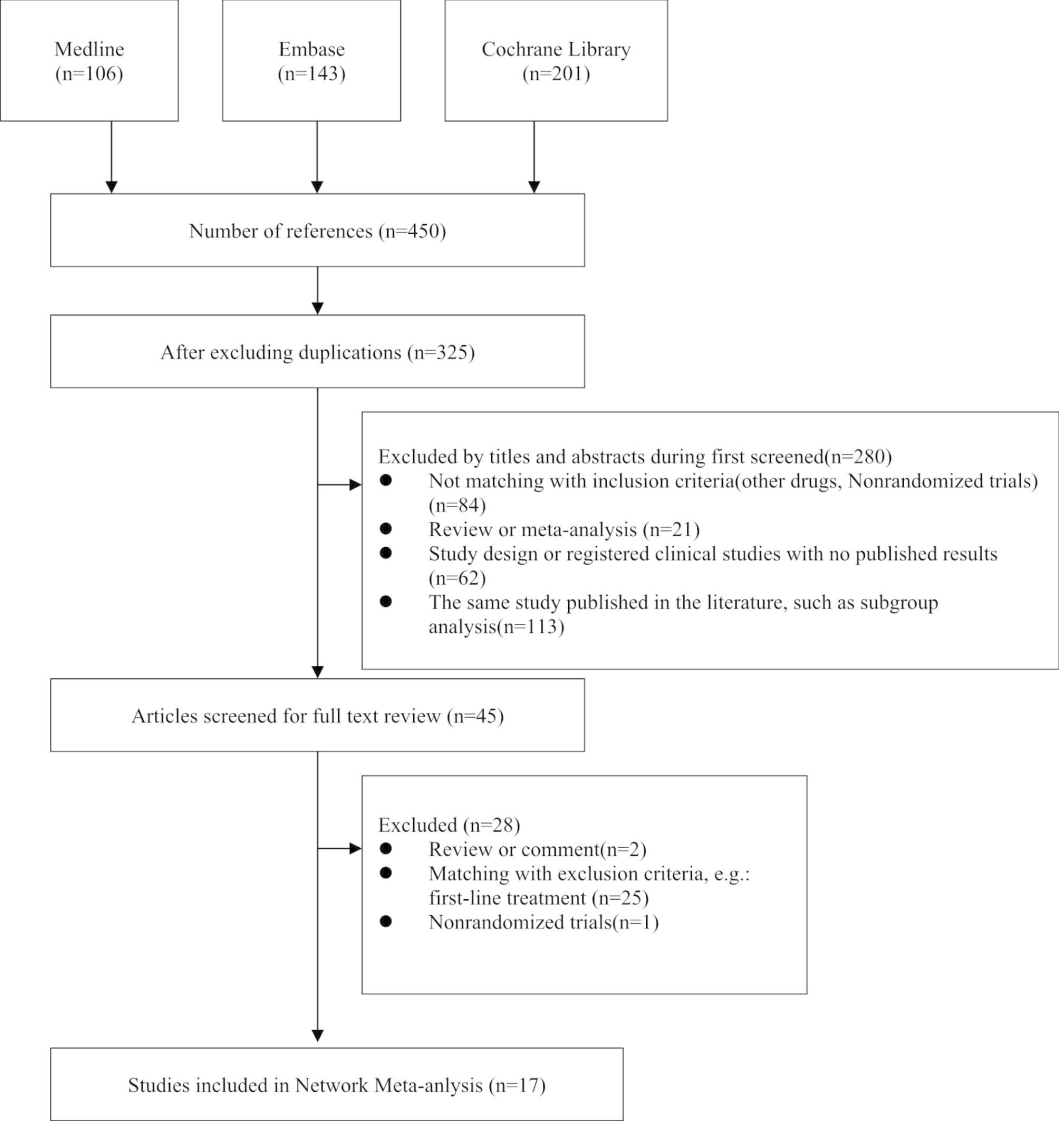
Flow chart for the identification of included studies

**Fig. 2:**
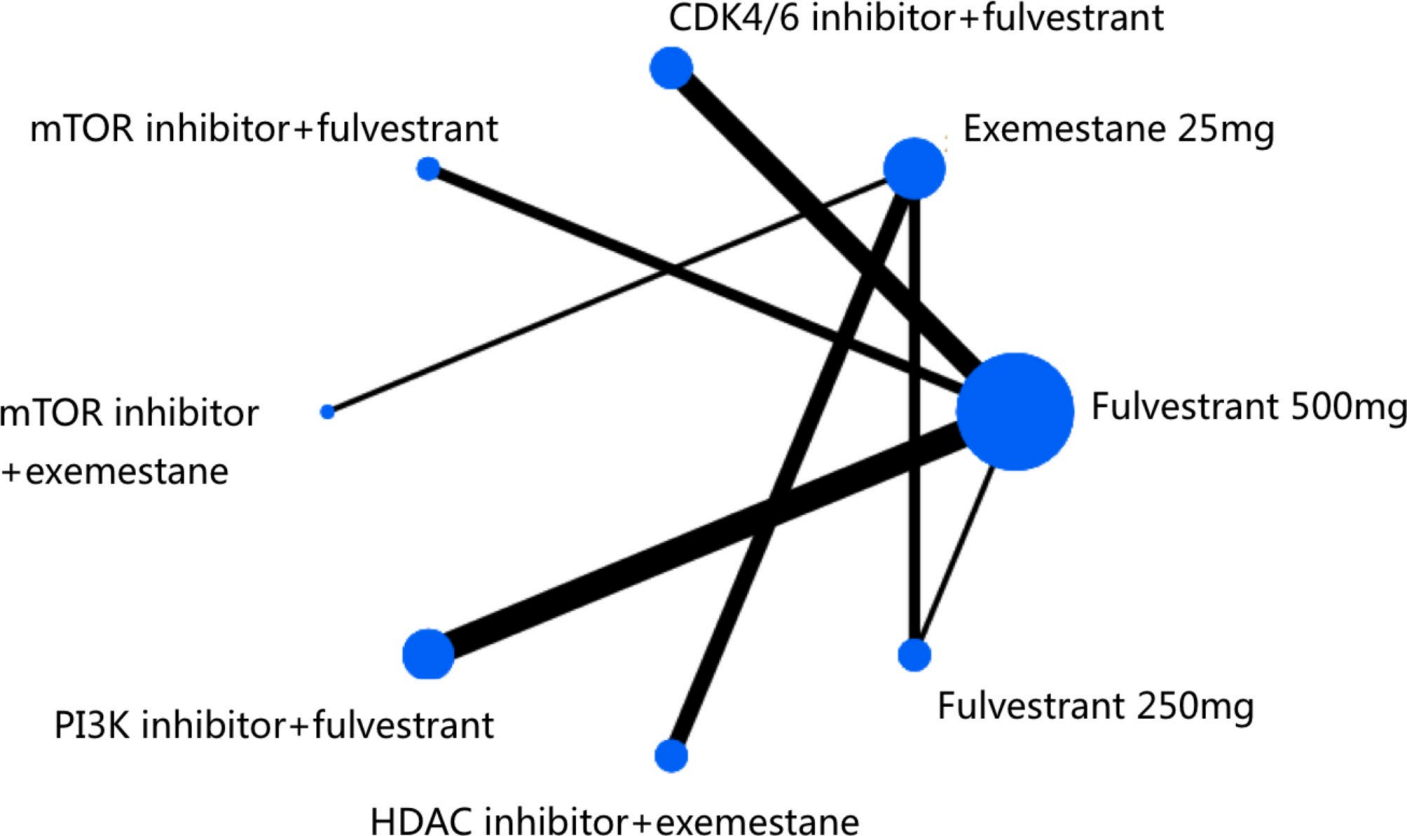
Network of the trials included in the analysis. The node size is proportional to the total number of patients in the regimen. The width of each line is proportional to the number of studies comparing the two regimens

After screening 450 citations (106 from Medline, 143 from Embase, and 201 from Cochrane Library), 17 studies with a total of 9,100 participants were included in the current study (Figs. 1 and 2). The characteristics of these studies are summarized in Table 1. Four of 17 trials compared CDK4/6 inhibitors plus fulvestrant with fulvestrant alone [10, 25–27], 4 trials investigated PI3K inhibitors plus fulvestrant [19, 28–30], 3 trials investigated mTOR inhibitors plus fulvestrant or exemestane [31–33], and 3 trials investigated HDAC inhibitors plus exemestane [20, 34, 35]. The remaining three studies (EFECT, SoFEA and CONFIRM) were designed to compare fulvestrant and exemestane in the network meta-analysis [36–38]. Most of the included studies were phase II or III clinical randomized controlled trials. All trials had a low risk of bias. No obvious evidence of publication bias was present based on a funnel plot (Supplemental Fig. 1).

**Table 1:** Study characteristics of the trials included in the network analysis

Author, year	Trail name	Pathway inhibitor	Phase Status	Intervention group(n)	Control group(n)	Patients	Outcomes*
Dennis J. Slamon, 2018	MONALEESA-3	CDK4/6 inhibitor	Phase III	ribociclib plus fulvestrant(n = 484)	placebo plus fulvestrant(n = 242)	Postmenopausal women and men with histologically and/or cytologically confirmed HR-positive/HER2-negative advanced breast cancer	1,2,3,5
Massimo Cristofanilli, 2016	PALOMA3	CDK4/6 inhibitor	Phase III	palbociclib plus fulvestrant(n = 347)	placebo plus fulvestrant(n = 174)	Confirmed hormone receptor-positive, HER2-negative metastatic breast cancer. Women aged 18 years or older of any menopausal status	1,2,3,4,5
George W. Sledge, 2017	MONARCH 2	CDK4/6 inhibitor	Phase III	abemaciclib plus fulvestrant(n = 446)	placebo plus fulvestrant(n = 223)	Women with hormone receptor-positive and human epidermal growth factor receptor 2-negative ABC who had progressed while receiving neoadjuvant or adjuvant endocrine therapy (ET)	1,2,3,4,5
Binghe Xu, 2021	DAWNA-1	CDK4/6 inhibitor	Phase III	dalpiciclib plus fulvestrant(n = 241)	placebo plus fulvestrant(n = 120)	Women of any menopausal status aged 18–75 years with pathologically confirmed hormone receptor-positive, HER2-negative locally advanced or metastatic breast cancer	1,2,3,4,5
José Baselga, 2012	BOLERO-2	mTOR inhibitor	Phase III	everolimus plus exemestane(n = 485)	placebo plus exemestane (n = 239)	Patients with hormone-receptor–positive advanced breast cancer who had recurrence or progression while receiving previous therapy with a nonsteroidal aromatase inhibitor.	1,2,3,4,5
Noah Kornblum, 2018	PrE0102	mTOR inhibitor	Phase II	everolimus Plus fulvestrant (n = 66)	placebo Plus fulvestrant (n = 65)	Postmenopausal women with ER-positive, human epidermal growth factor receptor 2–negative, AI-resistant metastatic breast cancer	1,2,3,4,5
Peter Schmid, 2019	MANTA	mTOR inhibitor	Phase II	fulvestrant plus vistusertib(n = 103), fulvestrant plus vistusertib(n = 98) fulvestrant plus everolimus(n = 65)	fulvestrant(n = 67)	Patients with estrogen receptor–positive breast cancer progressing after prior aromatase inhibitor treatment	1,2,3,4,5
F. André, E, 2019	SOLAR-1	PI3K inhibitor	Phase III	Cohort with PIK3CA-Mutated Cancer: alpelisib plus fulvestrant (n = 169)/Cohort without PIK3CA-Mutated Cancer: alpelisib plus fulvestrant (n = 115)	Cohort with PIK3CA-Mutated Cancer: placebo plus fulvestrant(n = 172)/Cohort without PIK3CA-Mutated Cancer: placebo plus fulvestrant(n = 116)	Men and postmenopausal women who had locally confirmed HR-positive, HER2-negative advanced breast cancer.	1,2,3,4,5
José Baselga, 2017	BELLE-2	PI3K inhibitor	Phase III	buparlisib plus fulvestrant(n = 576)	placebo plus fulvestrant(n = 571)	Postmenopausal women aged 18 years or older with histologically confirmed, hormone receptor-positive and human epidermal growth factor (HER2)-negative inoperable locally advanced or metastatic breast cancer whose disease had progressed on or after aromatase inhibitor treatment.	1,2,3,4,5
Angelo Di Leo, 2017	BELLE-3	PI3K inhibitor	Phase III	buparlisib(n = 289)	placebo(n = 143)	HER2-negative, locally advanced or metastatic breast cancer, who had relapsed on or after endocrine therapy and mTOR inhibitors.	1,2,4,5
Ian E Krop, 2016	FERGI	PI3K inhibitor	Phase II	Part 1: Pictilisib plus fulvestrant (n = 89)/Part 2:Pictilisib plus fulvestrant (n = 41)	Part 1: Placebo plus fulvestrant (n = 79)/Part 2:Placebo plus fulvestrant (n = 20)	Postmenopausal women aged 18 years or older with estrogen receptor-positive, HER2-negative breast cancer resistant to treatment with an aromatase inhibitor.	1,2,3,5
Zefei Jiang, 2019	ACE	HDAC inhibitor	Phase III	tucidinostat group(n = 244)	placebo group(n = 121)	Postmenopausal women with hormone receptor-positive, HER2-negative breast cancer, whose disease had relapsed or progressed after at least one endocrine therapy	1,3,4,5
Denise A. Yardley, 2013	ENCORE301	HDAC inhibitor	Phase II	entinostat plus exemestane(n = 64)	placebo plus exemestane(n = 66)	Postmenopausal women with ER + advanced breast cancer progressing on a nonsteroidal aromatase inhibitor	1,2,3,4,5
Roisin M. Connolly, 2021	E2112	HDAC inhibitor	Phase III	exemestane plus entinostat(n = 305)	exemestane plus placebo(n = 303)	Men or women with advanced HR-positive, HER2-negative breast cancer	1,2,3,4,5
Stephen Chia, 2008	EFECT	-	Phase III	fulvestrant(n = 351)	exemestane(n = 342)	Postmenopausal women with HR + advanced breast cancer progressing or recurring after nonsteroidal AI	1,3,4,5
Stephen R D Johnston, 2013	SoFEA	-	Phase III	fulvestrant plus anastrozole(n = 243)	fulvestrant plus placebo(n = 231)exemestane(n = 249)	Postmenopausal women with hormone-receptor-positive breast cancer	1,2,3,4,5
Angelo Di Leo, 2010	CONFIRM	-	Phase III	fulvestrant 500 mg(n = 362)	fulvestrant 250 mg(n = 374)	Postmenopausal women with estrogen receptor–positive advanced breast cancer	1,2,3,4,5

*1 PFS; 2 OS; 3 ORR; 4 CBR; 5 Safety PFS: progression-free survival; OS: overall survival; ORR: objective response rate; CBR, clinical benefit rate

### Network meta-analysis

A network meta-analysis comparing all combinations of individual medications was first performed (Supplement Table 1). Because medications in the same category did not differ, we pooled the data from different ones in the same category into one group in the present study.

The results of indirect comparisons using a network meta-analysis and the ORs are shown in Table 2, providing pairwise comparisons between each reagent in terms of PFS and ORR. For PFS, CDK4/6 inhibitor plus fulvestrant (OR 3.28, 95% CI: 2.12–5.07), mTOR inhibitor plus fulvestrant (OR 3.54, 95% CI: 1.35–9.34), mTOR inhibitor plus exemestane (OR 4.39, 95% CI: 1.79–10.81), and PI3K inhibitor plus fulvestrant (OR 2.12, 95% CI: 1.28–3.54) were superior to fulvestrant alone. Compared with placebo plus exemestane, PFS was significantly improved by the mTOR inhibitor plus exemestane and the HDAC inhibitor plus exemestane. In addition, the CDK4/6 inhibitor plus fulvestrant showed a superior effect compared with the PI3K inhibitor plus fulvestrant (OR 1.54, 95% CI: 1.07–2.23), while the mTOR inhibitor plus exemestane was superior to the HDAC inhibitor plus exemestane (OR 2.02, 95% CI: 1.25–3.28).

**Table 2:** Network meta-analysis of CDK4/6 inhibitors, PI3K/mTOR inhibitors and HDAC inhibitors for PFS and ORR

Fulvestrant 500 mg	0.87 (0.35,2.15)	0.40 (0.34,0.48)	0.37 (0.15,0.91)	0.30 (0.11,0.81)	0.62 (0.45,0.86)	0.61 (0.24,1.57)	1.33 (0.89,1.97)
1.23 (0.54,2.78)	Exemestane 25 mg	0.46 (0.18,1.16)	0.43 (0.12,1.51)	0.35 (0.23,0.51)	0.71 (0.27,1.86)	0.70 (0.53,0.93)	1.52 (0.67,3.42)
0.51 (0.38,0.69)	0.42 (0.18,1.00)	CDK4/6 inhibitor + fulvestrant	0.92 (0.38,2.28)	0.75 (0.27,2.03)	1.54 (1.07,2.23)	1.51 (0.58,3.95)	3.28 (2.12,5.07)
0.55 (0.29,1.04)	0.45 (0.16,1.27)	1.07 (0.53,2.16)	mTOR inhibitor + fulvestrant	0.81 (0.22,3.03)	1.67 (0.65,4.28)	1.63 (0.45,5.96)	3.54 (1.35,9.34)
0.15 (0.04,0.56)	0.12 (0.04,0.35)	0.28 (0.07,1.13)	0.26 (0.06,1.17)	mTOR inhibitor + exemestane	2.07 (0.73,5.82)	2.02 (1.25,3.28)	4.39 (1.79,10.81)
0.52 (0.36,0.75)	0.42 (0.17,1.04)	1.01 (0.63,1.62)	0.94 (0.45,1.96)	3.57 (0.88,14.53)	PI3K inhibitor + fulvestrant	0.98 (0.36,2.66)	2.12 (1.28,3.54)
0.78 (0.29,2.07)	0.63 (0.37,1.08)	1.51 (0.54,4.19)	1.40 (0.44,4.52)	5.35 (1.61,17.77)	1.50 (0.52,4.27)	HDAC inhibitor + exemestane	2.17 (0.92,5.13)
0.89 (0.49,1.60)	0.72 (0.41,1.28)	1.73 (0.90,3.33)	1.61 (0.67,3.83)	6.11 (1.81,20.64)	1.71 (0.85,3.43)	1.14 (0.52,2.50)	fulvestrant 250 mg

The results are presented as the OR and 95% CI for PFS (white quarter) and as the OR and 95% CI for ORR (green quarter)

For PFS, ORs that are lower than 1 favor the column-defining regimen. For ORR, ORs that are lower than 1 favor the row-defining regimen. The significance of values in blod red indicate that the ORs and the corresponding 95% CI have the significant difference

Compared with placebo plus fulvestrant, the ORR was significantly improved by CDK4/6 inhibitor plus fulvestrant (OR 0.51, 95% CI: 0.38–0.69), mTOR inhibitor plus exemestane (OR 0.15, 95% CI: 0.04–0.56), and PI3K inhibitor plus fulvestrant (OR 0.52, 95% CI: 0.36–0.75), but not mTOR inhibitor plus fulvestrant or HDAC inhibitor plus exemestane. Compared with placebo plus exemestane, the ORR was significantly improved by CDK4/6 inhibitor plus fulvestrant and mTOR inhibitor plus exemestane. In addition, the mTOR inhibitor plus exemestane was also superior to the HDAC inhibitor plus exemestane (OR 5.35, 95% CI: 1.61–17.77).

### SUCRA rankings

**Fig. 3:**
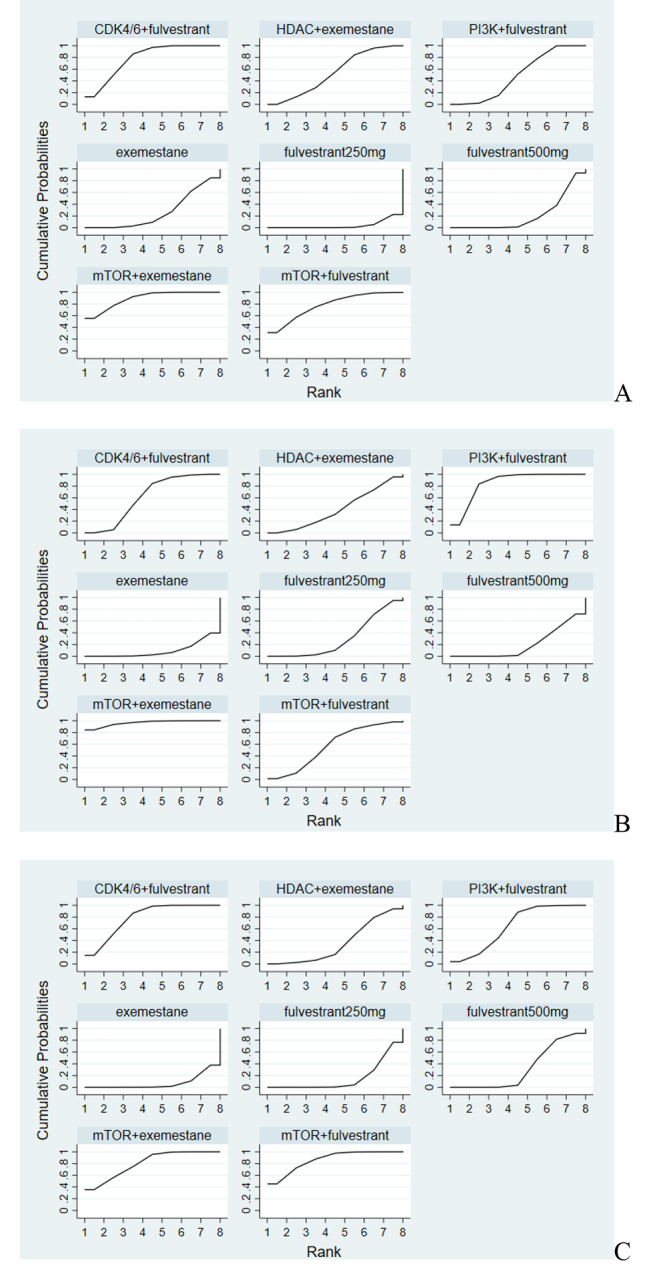
SUCRA ranking of each regimen in the network meta-analysis. (**A**) PFS; (**B**) ORR; (**C**) CBR.

In the network meta-analysis, the rankings of different combination regimens for outcomes in terms of PFS, ORR and CBR were expressed as SUCRA values (Fig. 3; Table 3). For PFS, mTOR inhibitor plus exemestane was the best regimen (SUCRA value 89.5%) and CDK4/6 inhibitor plus fulvestrant was the second optimal regimen (SUCRA value 77.8%), followed by mTOR inhibitor plus fulvestrant (SUCRA value 77.6%), HDAC inhibitor plus exemestane (SUCRA value 53.8%), and PI3K inhibitor plus fulvestrant (SUCRA value 49.4%). In terms of ORR, mTOR inhibitor plus exemestane was also the best regimen (96.3%), followed by PI3K inhibitor plus fulvestrant (84.7%), followed by CDK4/6 inhibitor plus fulvestrant (61.6%), mTOR inhibitor plus fulvestrant (57.1%), and HDAC inhibitor plus exemestane (40.1%). Regarding CBR, the SUCRA values were 80.3%, 78.8%, 86.1%, 64.5% and 35.3% for mTOR inhibitor plus exemestane, CDK4/6 inhibitor plus fulvestrant, mTOR inhibitor plus fulvestrant, PI3K inhibitor plus fulvestrant and HDAC inhibitor plus exemestane, respectively.

**Table 3:** SUCRA of each combination regimen in the network meta-analysis

Treatments	PFS	ORR	CBR
mTOR inhibitor plus exemestane	89.5	96.3	80.3
CDK4/6 inhibitor plus fulvestrant	77.8	61.6	78.8
mTOR inhibitor plus fulvestrant	77.6	57.1	86.1
HDAC inhibitor plus exemestane	53.8	40.1	35.3
PI3K inhibitor plus fulvestrant	49.4	84.7	64.5
Exemestane 25 mg	26.8	9.3	7.2
Fulvestrant 500 mg	21.0	20.3	32.1
Fulvestrant 250 mg	4.1	30.5	15.7

PFS: progression-free survival; ORR: objective response rate; CBR, clinical benefit rate

### Adverse effects

The most common grade 3 or 4 adverse events from the treatments in each trial are summarized in Table 4. The common AEs observed with CDK4/6 inhibitors plus endocrine are neutropenia, leukopenia, diarrhea, anemia, thrombocytopenia and lymphopenia. Notably, neutropenia was the most common AE in all CDK4/6 inhibitors plus endocrine studies. AEs reported in mTOR inhibitors plus endocrine studies include stomatitis, fatigue and asthenia, diarrhea, cough, pyrexia, and hyperglycemia. PI3K inhibitor combination therapy AEs with an incidence of at least 10% include neutropenia, leucopenia, thrombocytopenia, fatigue, decreased neutrophil count and hypophosphatemia. For HDAC combination therapy, the reported AEs include neutropenia, leukopenia, thrombocytopenia, fatigue, decreased neutrophil count and hypophosphatemia. The risks of drug-related severe adverse events (SAEs) were similar among all treatment regimens except for the mTOR inhibitor plus exemestane regimen, which induced more SAEs than the HDAC inhibitor plus exemestane regimen (OR 2.40; 95% CI: 1.40–4.10). The withdrawal rates were relatively low in the HDAC inhibitor plus fulvestrant (2.5–15.9%) and mTOR inhibitor plus fulvestrant (9.0-18.8%) groups and high in the PI3K inhibitor plus fulvestrant (9.5–39%) and HDAC inhibitor plus exemestane (11.0–64.0%) groups.

**Table 4:** Toxicity profile of treatments in each included trial

	Common grade ≥ 3 AEs with at least 5% incidence	Drug related SAE (%)	Withdrawal rate (%)
**MONALEESA-3**			
Ribociclib Plus Fulvestrant	Neutropenia 53.4%, Leukopenia 14.1%	11.2	4.3
Placebo Plus Fulvestrant		2.5	2.1
**PALOMA3**			
Fulvestrant Plus Palbociclib	Neutropenia 62%	9.6	4
Placebo Plus Fulvestrant	-	14.4	2
**MONARCH 2**			
Fulvestrant Plus Abemaciclib	Diarrhea 13.3%, neutropenia 26.5%, anemia 7.2%, Leukopenia 8.8%	8.8	15.9
Placebo Plus Fulvestrant	-	1.3	3.1
**DAWNA-1**			
Dalpiciclib Plus Fulvestrant	Neutropenia 84.2%, Leukopenia 62.1%, Thrombocytopenia 5.8%, Lymphopenia 5%	5.8	2.5
Placebo Plus Fulvestrant	-	6.7	3.3
**BOLERO-2**			
Exemestane Plus Everolimus	Stomatitis 8%, anemia 6%,	13.1	-
Exemestane	-	1.7	-
**PrE0102**			
Fulvestrant Plus Everolimus	Oral mucositis 11%, Fatigue 6%, Pneumonitis 6%	-	9
Fulvestrant Plus Placebo	Fatigue 5%	-	9
**MANTA**			
Fulvestrant Plus Daily Vistusertib	stomatitis 13.0%, rash 20.7%, infection 5.4%	-	17.8
Fulvestrant Plus Intermittent Vistusertib	asthenia 5.4%, diarrhea 5.4%	-	16.8
Fulvestrant Plus Everolimus	stomatitis 11.7%, asthenia 5.4%, diarrhea 5.4%, infection 6.7%	-	18.8
Fulvestrant		-	9.1
**SOLAR-1**			
Alpelisib Plus Fulvestrant	Hyperglycemia 36.6%, diarrhea 6.7%, rash 9.9%	34.9	9.5
Placebo Plus Fulvestrant	-	16.7	3.5
**BELLE-2**			
Buparlisib Plus Fulvestrant	Hyperglycemia < 16%, Increased ALT 26%, Increased AST 18%, rash < 9%, fatigue 5%, depression 5%, anxiety < 6%	23	39
Placebo Plus Fulvestrant	-	16	5
**BELLE-3**			
Buparlisib Plus Fulvestrant	Increased ALT 22%, Increased AST 18%, Hyperglycemia 13%, Hypertension 6%	22	-
Placebo Plus Fulvestrant	-	16	-
**FERGI**			
Pictilisib Plus Fulvestrant	Maculopapular rash 9%, diarrhea 8%, fatigue 8%, ALT concentration increased 5%, rash 7%	16	22
Placebo Plus Fulvestrant	-	13	5
**ACE**			
Tucidinostat	Neutropenia 51%, leucopenia < 19%, thrombocytopenia 27%, hypertriglyceridemia, hypokalemia < 7%, Increased γ-glutamyl transferase, 5%	21	64
Placebo	-	6	74
**ENCORE301**			
Exemestane Plus Entinostat	Fatigue 13%, Nausea 5%, Neutropenia 15%, Vomiting 5%	16	11
Exemestane Plus Placebo	-	12	2
**E2112**			
Entinostat	White blood cell decreased 6%, Neutrophil count decreased < 20%, Anemia < 8%, Hypophosphatemia < 14%	4.4	16
Placebo	-	5.1	8
**EFECT**			
Fulvestrant250	Injection-site pain 9.8%, hot flashes 8.8%, nausea 6.8%, fatigue 6.3%	1.1	2
Exemestane	Hot flashes 11.5%, fatigue 10%, nausea 7.5%, arthralgia 5.6%	0.6	2.6
**SoFEA**			
Fulvestrant250 + Anastrozole	-	14.8	2.8
Fulvestrant250	Fatigue 5%	22	3.4
Exemestane	Fatigue 5%	29	3.6
**CONFIRM**			
Fulvestrant250	-	7.2	2.2
Fulvestrant500	-	9.7	1.6

## Discussion

Adding CDK4/6, PI3K, mTOR and HDAC inhibitors to endocrine therapy has proven to be an effective second-line treatment strategy for patients with endocrine resistance. Compared to standard hormone therapies alone, the median progression-free survival nearly doubled and the proportion of patients achieving an overall response significantly improved in all pivotal trials of hormone therapies combined with CDK4/6 inhibitors, mTOR inhibitors, and PI3K inhibitors [9, 10, 31, 39–42]. However, direct comparisons of these combinations are lacking, and treatment decisions are difficult to make in real-world practice. Thus, we performed this network meta-analysis to provide more information on therapeutic regimens for clinicians. To our knowledge, this study is the first to indirectly compare the effectiveness of these novel agents in categories using a network meta-analysis. In terms of PFS, the CDK4/6 inhibitor plus fulvestrant showed a superior effect compared with the PI3K inhibitor plus fulvestrant, and the mTOR inhibitor plus exemestane was superior to the HDAC inhibitor. In addition, the mTOR inhibitor plus exemestane was also superior to the HDAC inhibitor for ORR. Importantly, the SUCRA values indicated that the combination of mTOR plus exemestane and CDK4/6 plus fluvestrant ranked first and second in a variety of regimens.

One previous network meta-analysis involving six trials with 4,063 patients indirectly compared the efficacy of palbociclib, abemaciclib and everolimus for restoring endocrine sensitivity and demonstrated that the combinations of palbociclib or abemaciclib with fulvestrant showed similar efficacies to everolimus plus exemestane in terms of PFS and ORR [43]. This finding indicates that the efficacies of CDK4/6 inhibition and mTOR blockade are similar. These results are similar to the findings of the present study. Another recently published network meta-analysis that included eight RCTs compared the efficacy and safety of three types of CDK4/6 inhibitors and five types of PI3K/AKT/mTOR inhibitors plus fulvestrant, but this study lacked HDAC inhibitor- and exemestane-based regimens [23]. Nevertheless, this meta-analysis showed that second-line treatment with three CDK4/6 inhibitors showed superior clinical efficacy compared to PI3K/AKT/mTOR inhibitors when combined with fulvestrant. However, our study provided additional information: mTOR inhibitors plus exemestane may achieve better outcomes than both CDK4/6 inhibitors plus fulvestrant and mTOR inhibitors plus fulvestrant.

The adverse effects (AEs) of drugs are also an important factor affecting the clinical choice of treatment. This study summarizes the AE rates of various agents, showing that all combination therapies were associated with a higher incidence of adverse events than endocrine therapy alone. However, further direct comparison of AEs between these combination therapies via RCTs is necessary.

The advantages of the present study are as follows. First, all the trials included in the analysis were well-designed RCTs of high quality with a low risk of bias. Second, the network analysis was performed according to the categories of the novel agents but not individual drugs, which improves the number of studies in each arm and reduces the risk of selection bias. Finally, CDK4/6 inhibitors, PI3K/mTOR inhibitors, and HDAC inhibitors were compared for the first time using a network meta-analysis with bridging studies.

One limitation of the present study is that the meta-analysis was not based on individual patient data, potentially influencing the validity of the results. Another limitation is that we could not analyze OS because of a lack of data, especially from EFECT and CONFIRM. In addition, the BELLE-3 study enrolled some patients with mTOR inhibitor resistance, which might affect the consistency of enrolled patients. Of note, the findings we presented were not for direct comparison.

## Conclusions

Based on the present network meta-analysis, we can conclude that mTOR inhibitor- and CDK4/6 inhibitor-based regimens demonstrated superior clinical efficacy and comparable safety profiles as second-line treatment in patients with HR-positive, HER2-negative advanced breast cancer compared to PI3K inhibitors and HDAC inhibitors.

### Electronic supplementary material

Below is the link to the electronic supplementary material.

Supplementary Material 1

## Data Availability

The datasets used and/or analysed during the current study available from the corresponding author on reasonable request.
