# Supplementary material for: CDK4/6 inhibitors, PI3K/mTOR inhibitors, and HDAC inhibitors as second-line treatments for hormone receptor-positive, HER2-negative advanced breast cancer: a network meta-analysis
Source: BMC Cancer. 2023 Aug 29;23:805. doi: 10.1186/s12885-023-11290-7 (PMC10463765; doi:10.1186/s12885-023-11290-7)
Supplement: Supplementary file 1 — Supplementary Material 1 [file 12885_2023_11290_MOESM1_ESM.docx]

Supplemental Table 1. Network meta-analysis of individual drugs of CDK4/6 inhibitors, PI3K/mTOR inhibitors and HDAC inhibitors for PFS.

| Fulvestrant  500 mg | 0.87 (0.35,2.15) | 0.46 (0.34,0.63) | 0.38 (0.26,0.55) | 0.42 (0.30,0.59) | 0.32 (0.20,0.51) | 0.37 (0.15,0.91) | 0.30 (0.11,0.81) | 0.84 (0.53,1.35) | 0.49 (0.39,0.61) | 0.77 (0.43,1.36) | 0.59 (0.21,1.66) | 0.62 (0.24,1.63) | 1.33 (0.89,1.97) |
| --- | --- | --- | --- | --- | --- | --- | --- | --- | --- | --- | --- | --- | --- |
| 1.14 (0.46,2.82) | Exemestane 25 mg | 0.53 (0.20,1.38) | 0.43 (0.16,1.15) | 0.48 (0.18,1.25) | 0.37 (0.13,1.01) | 0.43 (0.12,1.51) | 0.35 (0.23,0.51) | 0.97 (0.35,2.68) | 0.56 (0.22,1.42) | 0.88 (0.30,2.56) | 0.68 (0.42,1.11) | 0.71 (0.50,1.00) | 1.52 (0.67,3.42) |
| 2.17 (1.58,2.97) | 1.89 (0.73,4.92) | Ribociclib +fulvestrant | 0.82 (0.50,1.34) | 0.90 (0.57,1.44) | 0.70 (0.40,1.21) | 0.81 (0.32,2.07) | 0.65 (0.23,1.83) | 1.83 (1.04,3.23) | 1.06 (0.72,1.56) | 1.66 (0.86,3.20) | 1.29 (0.44,3.76) | 1.34 (0.48,3.71) | 2.87 (1.73,4.76) |
| 2.65 (1.81,3.86) | 2.31 (0.87,6.15) | 1.22 (0.75,2.00) | Palbociclib +fulvestrant | 1.10 (0.66,1.84) | 0.85 (0.47,1.54) | 0.99 (0.38,2.59) | 0.80 (0.28,2.29) | 2.24 (1.22,4.09) | 1.29 (0.83,2.01) | 2.03 (1.02,4.05) | 1.57 (0.53,4.69) | 1.64 (0.58,4.63) | 3.51 (2.03,6.07) |
| 2.40 (1.70,3.38) | 2.10 (0.80,5.50) | 1.11 (0.70,1.77) | 0.91 (0.54,1.51) | Abemaciclib +fulvestrant | 0.77 (0.44,1.36) | 0.90 (0.35,2.32) | 0.72 (0.26,2.05) | 2.03 (1.13,3.63) | 1.17 (0.78,1.77) | 1.84 (0.95,3.60) | 1.43 (0.48,4.21) | 1.49 (0.53,4.14) | 3.18 (1.89,5.37) |
| 3.11 (1.97,4.91) | 2.72 (0.99,7.47) | 1.44 (0.83,2.50) | 1.18 (0.65,2.13) | 1.30 (0.73,2.29) | Dalpiciclib +fulvestrant | 1.16 (0.43,3.15) | 0.94 (0.32,2.78) | 2.63 (1.37,5.06) | 1.52 (0.91,2.53) | 2.39 (1.15,4.98) | 1.85 (0.60,5.68) | 1.93 (0.66,5.61) | 4.13 (2.26,7.54) |
| 2.67 (1.10,6.47) | 2.34 (0.66,8.26) | 1.24 (0.48,3.16) | 1.01 (0.39,2.64) | 1.11 (0.43,2.88) | 0.86 (0.32,2.32) | Everolimus +fulvestrant | 0.81 (0.22,3.03) | 2.26 (0.83,6.15) | 1.30 (0.52,3.25) | 2.06 (0.72,5.90) | 1.59 (0.41,6.16) | 1.65 (0.45,6.13) | 3.54 (1.35,9.34) |
| 3.31 (1.24,8.86) | 2.89 (1.96,4.28) | 1.53 (0.55,4.30) | 1.25 (0.44,3.59) | 1.38 (0.49,3.91) | 1.06 (0.36,3.15) | 1.24 (0.33,4.65) | Everolimus +exemestane | 2.80 (0.94,8.33) | 1.62 (0.59,4.43) | 2.55 (0.82,7.95) | 1.97 (1.06,3.68) | 2.05 (1.22,3.46) | 4.39 (1.79,10.81) |
| 1.18 (0.74,1.90) | 1.03 (0.37,2.86) | 0.55 (0.31,0.96) | 0.45 (0.24,0.82) | 0.49 (0.28,0.88) | 0.38 (0.20,0.73) | 0.44 (0.16,1.21) | 0.36 (0.12,1.06) | Alpelisib +fulvestrant | 0.58 (0.34,0.97) | 0.91 (0.43,1.91) | 0.70 (0.23,2.18) | 0.73 (0.25,2.15) | 1.57 (0.85,2.90) |
| 2.05 (1.63,2.57) | 1.79 (0.71,4.54) | 0.95 (0.64,1.40) | 0.77 (0.50,1.20) | 0.85 (0.57,1.29) | 0.66 (0.40,1.09) | 0.77 (0.31,1.91) | 0.62 (0.23,1.70) | 1.73 (1.03,2.92) | Buparlisib +fulvestrant | 1.58 (0.85,2.92) | 1.22 (0.43,3.48) | 1.27 (0.47,3.42) | 2.72 (1.72,4.29) |
| 1.30 (0.73,2.31) | 1.14 (0.39,3.31) | 0.60 (0.31,1.16) | 0.49 (0.25,0.98) | 0.54 (0.28,1.06) | 0.42 (0.20,0.87) | 0.49 (0.17,1.40) | 0.39 (0.13,1.23) | 1.10 (0.52,2.31) | 0.63 (0.34,1.18) | Pictilisib +fulvestrant | 0.77 (0.24,2.51) | 0.81 (0.26,2.48) | 1.72 (0.86,3.46) |
| 1.68 (0.60,4.69) | 1.47 (0.90,2.39) | 0.78 (0.27,2.27) | 0.64 (0.21,1.90) | 0.70 (0.24,2.07) | 0.54 (0.18,1.66) | 0.63 (0.16,2.44) | 0.51 (0.27,0.95) | 1.42 (0.46,4.39) | 0.82 (0.29,2.35) | 1.29 (0.40,4.19) | Chidamide +exemestane | 1.04 (0.57,1.89) | 2.23 (0.87,5.74) |
| 1.62 (0.61,4.25) | 1.41 (1.00,2.00) | 0.75 (0.27,2.07) | 0.61 (0.22,1.73) | 0.67 (0.24,1.88) | 0.52 (0.18,1.51) | 0.60 (0.16,2.24) | 0.49 (0.29,0.82) | 1.37 (0.47,4.00) | 0.79 (0.29,2.13) | 1.24 (0.40,3.83) | 0.96 (0.53,1.75) | Entinostat +exemestane | 2.14 (0.89,5.18) |
| 0.75 (0.51,1.12) | 0.66 (0.29,1.48) | 0.35 (0.21,0.58) | 0.29 (0.16,0.49) | 0.31 (0.19,0.53) | 0.24 (0.13,0.44) | 0.28 (0.11,0.74) | 0.23 (0.09,0.56) | 0.64 (0.34,1.18) | 0.37 (0.23,0.58) | 0.58 (0.29,1.16) | 0.45 (0.17,1.16) | 0.47 (0.19,1.13) | Fulvestrant  250 mg |

Supplemental Fig. 1. Funnel plot of the trials included in the network meta-analysis
